# Evaluation on curative effects of adjuvant chemotherapy alone in treating with advanced endometrial carcinoma: A protocol for systematic review and meta-analysis

**DOI:** 10.1097/MD.0000000000028817

**Published:** 2022-03-04

**Authors:** Jie Jiang, Lin Chen, Yong Tian

**Affiliations:** 1 Department of Obstetrics and Gynecology, Central Hospital of Enshi Tujia and Miao Autonomous Prefecture, Enshi Clinical College of Wuhan University, Enshi, Hubei, China.

**Keywords:** advances stage, chemotherapy, curative effect, endometrial carcinoma

## Abstract

**Background:**

Endometrial carcinoma is classified as a gynecological cancer with high incidence. However, comparatively, only a small percentage of patients associated with it experience the condition progress to advanced disease or face recurring conditions. In the event where the condition progresses or recurs, the existing prognosis is poor and the most efficient form of treatment has not been established. Traditional methods to treat advanced endometrial carcinoma involves cytoreductive surgical intervention and radiation therapy, or chemotherapy, or a combination. Currently, there is controversy regarding the safest and most effective form of treatment. Therefore, the aim of conducting this protocol is to systematically review and provide meta-analyses on the curative effects of only using adjuvant chemotherapy to treat advanced endometrial carcinoma.

**Methods:**

A systematic search will be performed in 6 online-based databases, including WanFang, PubMed, Web of Science, EMBASE, and Cochrane Library, China National Knowledge Infrastructure databases. All related studies until December 22, 2021 will be considered in the search. Moreover, Google Scholar will be used as a source for grey literature. Two independent authors will screen the titles and abstracts. We will use the revised Cochrane risk of bias tool for performing an assessment of the risk of bias in randomized controlled trials. Additionally, Begg test statistics and Egger regression test will be employed to objectively detect the publication bias.

**Ethics and dissemination:**

Not required.

**OSF registration:**

10.17605/OSF.IO/JA48Q.

## 1 Introduction

In Western society, endometrial cancer is the most prevalent form of gynecologic malignancy. In 2020, there was an estimated 65,620 fresh cases and 12,590 fatalities.^[1]^ International Federation of Gynecology and Obstetrics staging specifies a 5-year survival rate, and most face favourable outcomes owing to timely diagnosis.^[2]^ However, those who suffer severe forms of the disease face severe outcomes, with 5-year survival rates corresponding to approximately 45% and 25% for stages III and IV, respectively.^[1,3]^ Besides the International Federation of Gynecology and Obstetrics stage, tumor grades and histologic types are prognostic factors. Females with locally advanced (stage III or IVA) endometrial carcinoma are classified as a heterogeneous cohort facing the risk of both local and systemic recurrence of the condition. Medical and pathologic influences on the recurrence risk include histologic subtype, the degree of abdominal and pelvic illnesses, nodal involvement, the efficacy of surgical resection, and the presence of extra nodal disease.^[4,5]^

Presently, surgery is the commonly employed method to treat endometrial cancer. Postsurgical therapy is customized according to distinct risk factors, including age of the patient, stage of the tumor, myometrial invasion depth, and histologic grade.^[6,7]^ Generally, adjuvant therapies encompass vaginal brachytherapy, pelvic external beam radiation therapy, chemotherapy, and modality therapy combines them.^[8]^ In spite of the higher toxicity and pelvic relapse associated with chemotherapy alone, randomized studies have reported that adjuvant chemotherapy is crucial in the treatment of advanced disease.^[9,10]^ An alternate approach involves combining chemotherapy and radiation therapy to control both systemic and local recurrences.^[11,12]^ However, no systematic analysis has been published on the curative effects of solely using adjuvant chemotherapy to treat advanced endometrial carcinoma. In this meta-analysis, the curative effects of solely adopting adjuvant chemotherapy to treat advanced endometrial carcinoma will be systematically evaluated to provide a strong evidence-based support for clinical applications.

## 2 Methods and analysis

The protocol is registered in the Open Science Framework (registration number, 10.17605/OSF.IO/JA48Q) on January 25, 2022. In the event where amendments are made to the protocol, the dates, changes, and rationales will be tracked in OSF. The reporting of this protocol follows the Preferred Reporting Items for Systematic Review and Meta-Analysis Protocols (PRISMA-P) 2015 statement.^[13]^

## 3 Criteria for considering studies for this review

### 3.1 Types of studies

Randomized controlled trials that satisfy the inclusion criteria will be considered for the present review.

### 3.2 Types of participants

Participants include females who have had a hysterectomy with an established histological diagnosis of endometrial cancer.

### 3.3 Types of interventions

As experimental groups, patients that received adjuvant chemotherapy can be considered. There are no restrictions on the duration of treatment and frequency. Adjuvant radiotherapy, different adjuvant chemotherapy regimen and adjuvant chemoradiation will be considered as controls.

### 3.4 Types of outcome measures

Overall survival is the primary outcome measure. Secondary outcome measures are progression-free survival, life standard, and adverse outcomes.

## 4 Search methods for identification of studies

### 4.1 Electronic searches

A systematic search will be performed in 6 online-based databases, including WanFang, PubMed, Web of Science, EMBASE, and Cochrane Library, China National Knowledge Infrastructure databases. All related studies until December 22, 2021 will be considered in the search. Moreover, Google Scholar will be used as a source for grey literature. We will promote the sensitivity of the strategies used for searching by incorporating keywords from related trials that were undetected in prior searches. We will not impose any language or publication constraints. The keywords and Medical Subject Headings listed below shall be used to search the e-databases: chemotherapy, endometrial carcinoma, randomized controlled trials.

### 4.2 Searching other resources

All related ongoing trials will be searched in clinicaltrials.gov. A manual search will be performed on the reference lists of eligible studies and use the ‘related citations’ feature of PubMed to find additional related trials.

## 5 Data collection and analysis

### 5.1 Selection of studies

A pair of authors will perform an independent assessment of the identified trials to determine their suitability for inclusion.

### 5.2 Data extraction and management

A pair of authors will conduct an independent extraction of data and evaluate the published papers in line with the selection criteria. In cases of missing/incomplete data, we will attempt to contact the corresponding authors to obtain the relevant information. The extracted data will be related to study design and methods, participants’ demographic characteristics, and details of adjuvant chemotherapy interventions, control interventions, and outcome measures.

### 5.3 Assessment of risk of bias in included studies

The Jadad score^[14]^ will be the baseline for evaluating the quality of the selected studies. The evaluation will involve generating random sequences, randomization, implementing the blind methods by subjects and scholars, and the withdrawal and withdrawal criteria. The first 3 criteria will be scored according to 3 levels: appropriate, unclear, and inappropriate.

### 5.4 Measures of treatment effect

We will use the hazard ratio for comparing the risk of mortality or progression of disease between the treatment cohort and the controls in time-to-event data. Meanwhile, we will use the risk ratio for dichotomous outcomes (adverse events).

### 5.5 Assessment of heterogeneity

The heterogeneity among studies shall be quantified using the *I* ^2^ statistic, where considerable heterogeneity is indicated by *I* ^2^ greater than 75%. Accordingly, when *I* ^*2*^ > 75%, results from each article shall be reported descriptively rather than combining them using meta-analysis.

### 5.6 Assessment of reporting biases

Begg test statistics and Egger regression test will be employed to objectively assess the publication bias. To visualize the publication bias, Egger publication bias plot will be employed to present corresponding graphs. If *P* ≤ .05, it will be considered as a statistically significant publication bias.

### 5.7 Sensitivity analysis

Based on the criteria listed below, we will perform a sensitivity analysis to ascertain the robustness of the conclusions in the review, the criteria is as follows: sample size, heterogeneity qualities, and statistical model (random-effects or fixed-effects model).

## 6 Discussion

The present systematic review will be conducted to evaluate the curative effects of solely administering adjuvant chemotherapy to treat advanced endometrial carcinoma. To the best knowledge of the authors, no previous systematic review and meta-analysis has been reported in this particular area of study. Hence, it is critical and urgent to conduct this study to further investigate the curative effects of solely administering adjuvant chemotherapy as a treatment method for acute endometrial carcinoma in females.

In the current systematic review, we will obtain the related literature with no language constraint. Accordingly, all potential trials related to the use of adjuvant chemotherapy to treat advanced stages of endometrial carcinoma shall be considered to ensure that all related trials will be considered. The results of this analysis will summarize the existing evidence related to the curative effects associated with the administration of adjuvant chemotherapy to treat patients in progressive stages of endometrial carcinoma. The evidence can also act as a useful reference for health policymakers and clinical practitioners.

## Author contributions

**Conceptualization:** Jie Jiang, Lin Chen, Yong Tian.

**Data curation:** Jie Jiang, Lin Chen, Yong Tian.

**Formal analysis:** Jie Jiang, Lin Chen, Yong Tian.

**Funding acquisition:** Yong Tian.

**Investigation:** Jie Jiang, Lin Chen.

**Methodology:** Jie Jiang, Yong Tian.

**Project administration:** Jie Jiang.

**Resources:** Lin Chen, Yong Tian.

**Software:** Jie Jiang.

**Validation:** Jie Jiang, Lin Chen, Yong Tian.

**Visualization:** Jie Jiang, Lin Chen, Yong Tian.

**Writing – original draft:** Jie Jiang, Lin Chen, Yong Tian.

**Writing – review & editing:** Yong Tian.
